# STINGAllo: a web server for high-throughput prediction of allosteric site-forming residues using internal protein nanoenvironment descriptors

**DOI:** 10.1093/bib/bbaf424

**Published:** 2025-08-21

**Authors:** Folorunsho Bright Omage, José Augusto Salim, Ivan Mazoni, Inácio Henrique Yano, Jorge Enrique Hernández González, Poliana Fernanda Giachetto, Ljubica Tasic, Raghuvir Krishnaswamy Arni, Goran Neshich

**Affiliations:** Computational Biology Research Group, Embrapa Digital Agriculture, Av. André Tosello, 209, Barão Geraldo, Campinas, SP, CEP 13083-886, Brazil; Biological Chemistry Laboratory, Department of Organic Chemistry, Institute of Chemistry, University of Campinas (UNICAMP), Rua Josué de Castro, s/n – Cidade Universitária “Zeferino Vaz”, Barão Geraldo, Campinas, SP, CEP 13083-861, Brazil; Department of Plant Biology, Institute of Biology, University of Campinas (UNICAMP), Rua Monteiro Lobato, 255 – Cidade Universitária “Zeferino Vaz”, Barão Geraldo, Campinas, SP, CEP 13083-862, Brazil; Computational Biology Research Group, Embrapa Digital Agriculture, Av. André Tosello, 209, Barão Geraldo, Campinas, SP, CEP 13083-886, Brazil; Computational Biology Research Group, Embrapa Digital Agriculture, Av. André Tosello, 209, Barão Geraldo, Campinas, SP, CEP 13083-886, Brazil; Multiuser Center for Biomolecular Innovation, Institute of Biosciences, Humanities and Exact Sciences, São Paulo State University (UNESP), Rua Cristóvão Colombo, 2265, Jardim Nazareth, São José do Rio Preto, SP, CEP 15054-000, Brazil; Computational Biology Research Group, Embrapa Digital Agriculture, Av. André Tosello, 209, Barão Geraldo, Campinas, SP, CEP 13083-886, Brazil; Biological Chemistry Laboratory, Department of Organic Chemistry, Institute of Chemistry, University of Campinas (UNICAMP), Rua Josué de Castro, s/n – Cidade Universitária “Zeferino Vaz”, Barão Geraldo, Campinas, SP, CEP 13083-861, Brazil; Multiuser Center for Biomolecular Innovation, Institute of Biosciences, Humanities and Exact Sciences, São Paulo State University (UNESP), Rua Cristóvão Colombo, 2265, Jardim Nazareth, São José do Rio Preto, SP, CEP 15054-000, Brazil; Computational Biology Research Group, Embrapa Digital Agriculture, Av. André Tosello, 209, Barão Geraldo, Campinas, SP, CEP 13083-886, Brazil

**Keywords:** allosteric regulation, allosteric site prediction, internal protein nanoenvironment (IPN), machine learning, allosteric site-forming residues (AFRs), per-residue classification, STING most relevant descriptors for IPNs

## Abstract

Allosteric regulation is essential for modulating protein function and represents a promising target for therapeutic intervention, yet the complex dynamics of the protein nanoenvironment hinder the reliable identification of allosteric sites. Traditional pocket-based predictors miss $\sim $18% of experimentally confirmed sites that lie outside surface invaginations. To overcome this limitation, we developed STINGAllo, an interactive web server that introduces a residue-centric machine-learning model. Using 54 optimized internal protein nanoenvironment descriptors, STINGAllo predicts allosteric site-forming residues at single-residue resolution. By integrating hydrophobic interaction networks, local density, graph connectivity, and a unique “sponge effect” metric, STINGAllo detects allosteric sites independently of surface geometry, including concave pockets, flat surfaces, or even cryptic regions. It achieves a success rate of $\sim $78% on benchmark datasets, substantially outperforming existing methods with a 60.2% overall success rate compared with 21.1%–24.2% for contemporary pocket-based predictors. Our analysis further reveals that nearly 52.7% of unique proteins in the Protein Data Bank [(PDB); 119 851 entries, 14 November 2024] contain at least one chain with a predicted allosteric site. STINGAllo accepts protein structures via PDB identifiers or custom uploads, provides interactive 3D visualization of predicted pockets, and supports integration into computational pipelines through a RESTful application programming interface. Overall, STINGAllo bridges advanced computational prediction with user-friendly design, offering a robust tool expected to deepen understanding of protein regulation and accelerate allosteric drug discovery. The server is freely accessible at https://www.stingallo.cbi.cnptia.embrapa.br/.

## Introduction

Allostery is a fundamental regulatory mechanism in which a molecule binding at one site of a protein influences activity at a distant site. This mechanism underlies many critical biological processes and is often described as the “second secret of life” due to its ubiquity and importance in molecular biology [1]. Allosteric regulation allows proteins to fine-tune their activity in response to cellular signals, and dysregulation of allostery is implicated in various diseases [2]. Importantly, allosteric sites have emerged as attractive targets in drug discovery. Because allosteric modulators bind outside of the active site, they can modulate protein function without completely abolishing it. This often leads to greater specificity and fewer side effects in allosteric drugs [3]. Many orthosteric drugs bind to highly conserved active sites, a characteristic that risks off-target effects on homologous proteins. Allosteric modulators, in contrast, typically target less-conserved surface regions, which allows for greater selectivity. Indeed, allosteric drugs can either enhance or inhibit protein activity and can even be used in conjunction with orthosteric drugs for synergistic effects [4]. These advantages have spurred growing interest in identifying and characterizing allosteric sites on proteins for therapeutic intervention.

Despite their significance, allosteric sites are often challenging to detect. Allosteric mechanisms are diverse and remain elusive in many proteins, partly because identifying the potential allosteric pockets or residues experimentally is difficult [5]. Unlike active sites, which can sometimes be inferred from substrate analogs or sequence conservation, allosteric sites are often located far from the active site and are not readily apparent from the static structure alone. A key aspect of this challenge involves cryptic pockets: transient binding sites that are closed or disordered in the ligand-free state but become structured and accessible through protein dynamics or upon ligand binding [6, 7]. The discovery and characterization of these sites have opened a new frontier in drug discovery, providing a powerful strategy to develop modulators for targets previously deemed “undruggable” [8]. Classical examples underline this challenge. For instance, hemoglobin’s well-known binding site for the effector 2,3-bisphosphoglycerate (2,3-BPG) is located in a cavity not obvious in the oxygen-bound state. In protein tyrosine phosphatase 1B, a flexible helix occludes a distal pocket in the ligand-free structure, making the allosteric site invisible to standard pocket-detection algorithms until the helix moves [7]. These examples highlight why computational prediction methods are invaluable, as they can analyze subtle features of protein structure that might hint at allostery, even before an allosteric ligand is known.

### Computational landscape of allosteric site prediction

Computational strategies for identifying allosteric sites fall into four complementary categories (Supplementary Table S1). First, *static pocket-geometry* examines a single conformation, scoring surface cavities with geometric and physicochemical descriptors [3]. Second, *dynamics-driven* methods incorporate conformational motion, through normal-mode analysis or fully atomistic molecular dynamics (MD)/Markov-state modeling, to reveal transient or cryptic pockets [1]. This approach can deliver atom-level detail, albeit at a higher computational cost. Third, ‘hybrid machine-learning frameworks’ integrate geometry, energetics, evolutionary conservation and, where available, dynamic information in ensemble or deep-neural-network architectures to boost predictive accuracy [5]. Finally, *residue-centric network models* recast the protein as a graph, ranking nodes by their communication centrality to pinpoint allosteric hotspots [9]. Within this landscape, our residue-level predictor, STINGAllo, extends the network paradigm with a rich set of internal protein nanoenvironment (IPN) descriptors [10, 11].

To address the multifaceted nature of allostery, we developed STINGAllo, a machine learning model that adopts a residue-centric strategy; instead of scoring entire pockets, it classifies each residue in a protein structure as allosteric or not based on a rich set of per-residue descriptors characterizing its *IPN*. In our recent study, we introduced STINGAllo and demonstrated its performance in identifying AFRs on a benchmark dataset [12]. The model leveraged over 1200 structural and physicochemical features (descriptors) derived from the IPNs (capturing properties, such as local flexibility, secondary structure context, hydrogen bonding, hydrophobic packing, electrostatic potentials, graph centrality measures, etc.), which were curated from the STING database. Through correlation-based filtering and statistical tests, this set of descriptors was distilled to 54 most informative features. These features included unique descriptors like the “sponge effect,” which describes how a residue’s environment absorbs structural perturbations. Other key features included the distance from the protein’s geometric center, hydrophobic interaction counts, localized electrostatic potentials, and graph-theoretic scores indicating a residue’s importance in intramolecular communication networks. Using this tailored feature set, we trained the ensemble classifiers and ultimately selected a high-performing CatBoost gradient-boosted decision tree model as the core of STINGAllo. This approach achieved encouraging results. For example, on a test set of 91 protein chains with known allosteric sites, STINGAllo obtained a distance to center (DCC) success rate of 78%. This means that, in most cases, the predicted cluster of AFRs fell within the known allosteric pocket region. The model also achieved an overall per-residue classification F1 score of 0.64 (Matthews correlation coefficient 0.64). These metrics are on par with, and in some aspects exceed, those of contemporary methods, validating the effectiveness of STINGAllo. Perhaps more importantly, the model’s per-residue prediction scheme provides fine-grained insight; instead of simply highlighting a region, STINGAllo pinpoints which specific residues are likely involved in allosteric modulation. Such granularity can aid experimentalists in designing site-directed mutagenesis studies to validate allosteric hotspots.

While the prior publication [12] of STINGAllo described the model and its validation in detail, it is crucial to make this tool readily available to the broader scientific community. Many biologists and bioinformaticians who could benefit from allosteric site predictions may not have the resources or expertise to run complex machine-learning pipelines on structural data. To maximize impact, we have developed the STINGAllo Web Server. It is a free and public application allowing users to run predictions via a simple interface. We also provide an accompanying RESTful application programming interface (API) for programmatic access. This paper serves as an introduction and technical note for the STINGAllo web server and API, clearly distinguishing itself from the previous model focus study. Here, we focus on the implementation, features, and use cases of the web server, and provide guidance on how it can be utilized in research workflows. We also position STINGAllo in the context of existing tools, highlighting unique features, and comparative advantages. By presenting multiple case studies, we illustrate how the web server can handle diverse scenarios, from well-known allosteric proteins to novel structures, and how the results can be interpreted. Overall, this resource is intended to bridge the gap between advanced computational methods and practical, user-friendly applications, empowering researchers to explore allostery in their proteins of interest with ease and confidence.

## Materials and methods

**Figure 1:**
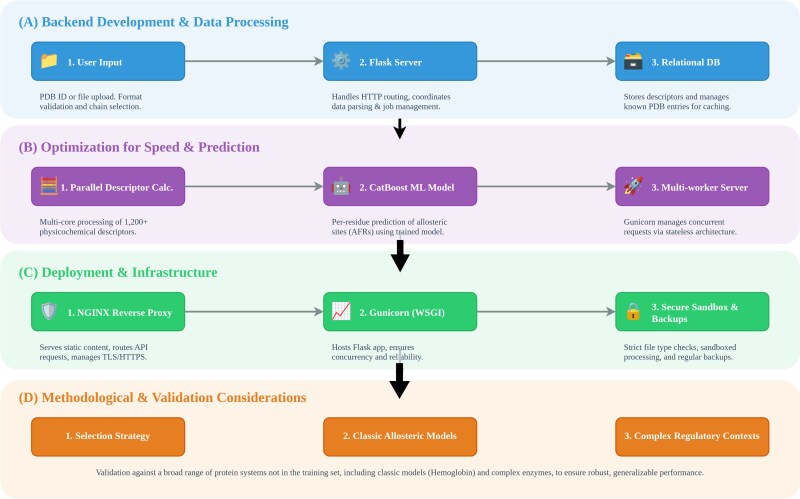
Overview of the STINGAllo methodology in four components: (A) backend development, including input validation, a Flask-based server, a relational database, and a CatBoost model for per-residue allosteric site prediction; (B) optimization for speed and scalability through parallelized descriptor calculations, batch CatBoost predictions, and a multi-worker architecture; (C) deployment and infrastructure with an NGINX reverse proxy, Gunicorn workers, sandboxed uploads, and a MySQL database; and (D) methodological considerations involving the selection of diverse protein systems, from classic allosteric models to complex regulatory contexts, to demonstrate broad applicability.

Figure 1 illustrates the computational pipeline of STINGAllo, from data input to allosteric site prediction. The web server and its underlying methodology are described in the following subsections.

### The STINGAllo prediction model: an overview

The STINGAllo web server is built upon a machine learning model designed to predict AFRs on a per-residue basis. The core of the predictor is a CatBoost gradient-boosted decision tree model, which was trained and validated as detailed in our previous work [12]. The model leverages a rich feature set of over 1200 structural and physicochemical descriptors that characterize the IPN of each residue. These descriptors, curated from the STING database [10, 11], capture a wide array of properties, including solvent accessibility, electrostatic potential, hydrophobic interaction networks, local secondary structure, and graph-theoretical connectivity metrics. Following a rigorous feature selection process, the final model utilizes the 54 most informative descriptors for classification. This work focuses on the implementation and utility of the web server that makes this predictive model accessible to the scientific community.

### Web server and application programming interface implementation

#### System architecture

The STINGAllo web server is implemented as a Flask-based Python web application and deployed on a Linux server. An NGINX reverse proxy manages incoming traffic, serving static content directly, and forwarding dynamic requests to a Gunicorn application server, which runs multiple worker processes to handle concurrent user sessions. The backend interfaces with a MySQL relational database that stores pre-calculated descriptor sets for all Protein Data Bank (PDB) entries, enabling rapid retrieval and prediction. The frontend provides an interactive user experience, featuring a 3D molecular viewer built with modern WebGL-based libraries to visualize protein structures and highlight predicted AFRs.

#### Input processing and prediction workflow

STINGAllo accepts protein structures via two main routes:

(1) **PDB identifier**: When a user provides a PDB ID, the system validates the identifier, retrieves the corresponding pre-computed feature set from the database. This workflow is highly optimized, typically returning results within seconds.
(2) **User-uploaded PDB file**: For custom structures, the server first validates the PDB file format. It then initiates a *de novo* calculation pipeline to generate the complete set of over 1200 IPN descriptors for each residue. This feature matrix is then used for prediction.

For both pathways, if a structure contains multiple chains, the user is prompted to select a single chain for analysis. The system classifies residues using the pre-trained CatBoost model.

#### RESTful application programming interface

To facilitate high-throughput analysis and integration into computational pipelines, we provide a RESTful API. The API allows programmatic access to pre-computed predictions for any structure in the PDB. Clients can make HTTP GET requests to the base endpoint:

https://www.stingallo.cbi.cnptia.embrapa.br/api/afrs

A valid request requires two query parameters: pdb_id and chain_id. For example:

curl ``https://.../api/afrs?pdb_id=1A01&chain_id=A''

A successful query returns a JSON object containing the list of predicted AFRs. The API is designed to be simple and lightweight, enabling easy integration with scripting languages like Python or command-line tools like curl. Full documentation and examples are available on the server’s website.

### Performance, scalability, and security

To ensure a responsive user experience, the STINGAllo server employs several optimization strategies. Descriptor calculations for new structures are parallelized, and server-side caching is used to store recent results, minimizing redundant computations. The multi-worker Gunicorn architecture allows the server to handle multiple requests simultaneously, and its stateless design supports horizontal scaling.

Security is a priority. All data transmission occurs exclusively over HTTPS. User-uploaded files are handled in a secure, sandboxed environment and are automatically deleted after the analysis is complete. The system incorporates robust file validation, defenses against common web vulnerabilities like SQL injection and cross-site scripting, and rate limiting at the NGINX level to ensure data integrity and system availability.

### Validation strategy

#### Benchmarking methodology

The underlying STINGAllo model was quantitatively benchmarked against other contemporary predictors, primarily the PASSer server [5, 13], as detailed in [12]. The benchmark was conducted on an independent test set of 91 protein chains from the ASBench database, which were not used during model training. The primary evaluation metric was the DCC success rate, which defines a successful prediction as one where the geometric center of the predicted AFRs cluster is within 4 Å of the geometric center of the experimentally confirmed allosteric site. This metric provides a robust measure of a predictor’s ability to correctly localize the allosteric pocket.

#### Case study selection

The protein systems chosen for the case studies in this manuscript were selected to represent a diverse range of well-characterized allosteric mechanisms, from classical models like hemoglobin to more complex examples of feedback inhibition and dimer-interface regulation. Crucially, none of the proteins featured in these case studies were part of the original training datasets used to develop the STINGAllo model. This ensures that their analysis serves as an unbiased, real-world evaluation of the server’s predictive performance and generalizability.

For clarity, we provide the following key definitions used throughout this work:

- **AFRs**: Residues that undergo significant structural changes [specifically, a loss of accessible surface area (LASA)] upon binding of the ligand to an allosteric site, according to the LASA criterion [12]. These residues physically contribute to the formation or stabilization of the allosteric binding pocket.
- **Allosteric residues**: A broader term encompassing all residues involved in allosteric communication pathways, including AFRs and residues participating in signal transmission between allosteric and orthosteric sites.

## Results

### STINGAllo database and web server overview

The STINGAllo Database currently comprises predictions for 227 316 unique PDB structures, spanning a wide variety of protein families and functions (based on data from Research Collaboratory for Structural Bioinformatics/PDB as of 14 November 2024). To accommodate the influx of new PDB entries, an automated update procedure is in place. For each structure, AFRs are predicted on a per-chain basis. Our analysis indicates that 119 851 unique PDB IDs (52.7% of the database) contain at least one chain with a valid AFRs prediction, whereas 83 383 unique PDB IDs (36.7%) yielded no AFRs predictions. The remaining entries consist of structures with incomplete descriptors or non-protein macromolecules. Table 1 summarizes this breakdown.

**Table 1:** Unique PDB IDs in the STINGAllo database by prediction status, categorized according to the best available chain prediction outcome.

Category	Unique PDB count^*^	Percentage (%)	Description
**a**	119 851	52.7	At least one chain with a valid AFRs prediction
**b**	83 383	36.7	No chain with an AFRs prediction
**c**	18 757	8.3	All chains with missing/incomplete descriptors
**d**	5325	2.3	Non-protein entries (e.g. RNA)
**Total**	227 316	100	Total unique PDB IDs processed

Category: **a**, valid AFRs prediction; **b**, no prediction; **c**, incomplete descriptors; **d**, non-protein entry. ^*^ As of 14 November 2024.

**Figure 2:**
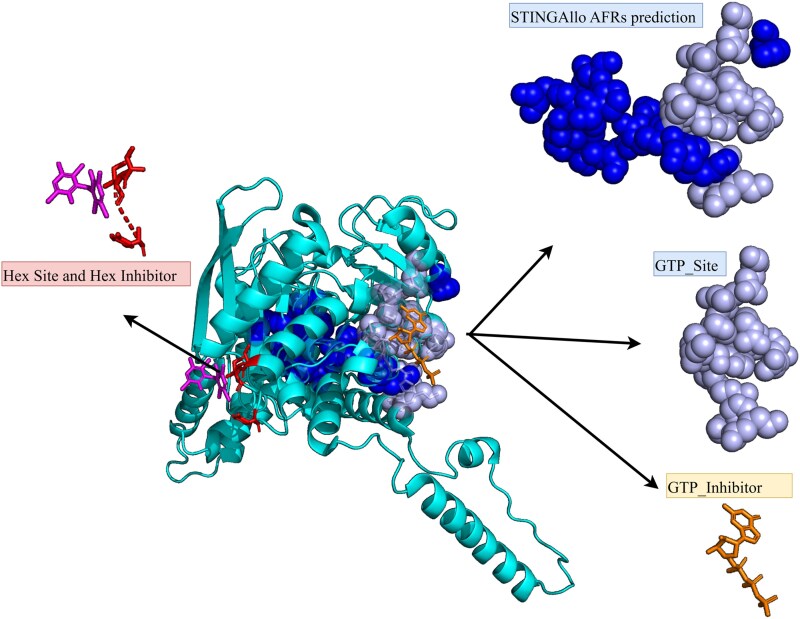
Structure of *T. thermophilus* glutamate dehydrogenase (PDB: 3ETE chain A) showing STINGAllo-predicted AFRs together with the hexachlorophene and GTP allosteric sites, illustrating accurate prediction of all 11 residues in the GTP site.

**Figure 3:**
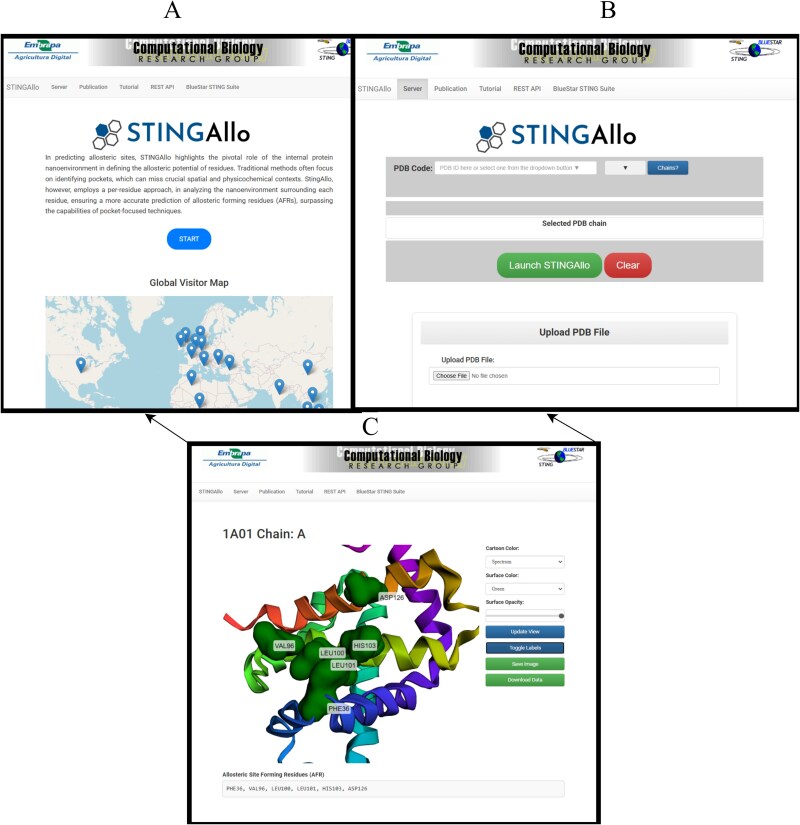
Overview of the STINGAllo web interface, showing the main landing page with a summary of the prediction approach, the user input section for specifying a PDB code and chain or uploading a file, and the interactive 3D viewer displaying predicted AFRs with adjustable visualization settings and downloadable results.

The web server, accessible at https://www.stingallo.cbi.cnptia.embrapa.br/, provides an intuitive interface for these prediction. To demonstrate how STINGAllo predictions integrate with known structural features, we highlight the *Thermus thermophilus* glutamate dehydrogenase structure (PDB ID: 3ETE chain A) as shown in Fig. 2. In this structure, the well-characterized catalytic site is absent; instead, two distinct allosteric regulatory sites are occupied by hexachlorophene (Hex_Site) and GTP (GTP_Site). STINGAllo correctly predicts all 11 residues constituting the GTP_Site (Ile212, Ser213, Arg217, Leu257, His258, Arg261, Tyr262, Arg265, Glu292, Lys446, and His450), a known inhibitory site [15], showcasing the predictor’s accuracy. Fig. 3 presents an overview of the STINGAllo web interface. (A) The main landing page features a synopsis of STINGAllo’s approach to allosteric site prediction and an interactive global visitor map. (B) The user input section allows specification of a PDB code and chain or the upload of a custom PDB file. (C) An example of the interactive 3D viewer displays results for hemoglobin (PDB ID: 1A01), with predicted AFRs highlighted in dark green surface representation. Users can modify visualization settings, toggle chains or residue labels, and download the prediction data using the controls on the right panel.

### Quantitative comparison with existing servers

A critical step in validating any new prediction server is to benchmark it against existing tools. While this web server paper focuses on the tool’s implementation and utility, the underlying STINGAllo model was rigorously benchmarked against other predictors as part of its development, with the full analysis detailed in our recent publication [12]. Here, we summarize the key quantitative comparisons to directly address how STINGAllo performs relative to other established servers.

Our comparative analysis focused on the PASSer server, a contemporary and widely used tool for allosteric site prediction. The rationale for this choice is two-fold. First, many earlier predictors (e.g. Allosite and AlloPred) are “pocket-based,” meaning their performance is fundamentally tied to the ability of algorithms like FPocket to correctly identify surface cavities. As we demonstrated in [12], this premise is a significant limitation, as $\sim $18% of experimentally verified AFRs in the ASBench dataset are not located within any pocket identified by FPocket. Such sites, which may be on flat surfaces or in cryptic locations, are systematically missed by pocket-based methods. STINGAllo’s “residue-centric” approach avoids this dependency, allowing it to identify AFRs regardless of their pocket context. Second, comparing against a modern tool like PASSer with available API to connect and which earlier studies have shown to perform better than many earlier predictors (e.g. Allosite and AlloPred) [5, 13] provides a more relevant assessment of state-of-the-art performance.

The primary metric for comparison was the DCC success rate, which measures whether the geometric center of the predicted AFRs is within 4 Å of the center of the experimentally known allosteric site. A lower DCC value indicates a more accurate prediction. The benchmark was performed on the 91-chain test set from ASBench, which was not used for training STINGAllo. The results, summarized in Table 2, clearly demonstrate STINGAllo’s superior performance.

**Table 2:** Summary of comparative performance of STINGAllo against PASSer models on the ASBench test set, with success defined as a DCC value $\leq $ 4 Å and data summarized from [12].

Predictor	Overall DCC	DCC success rate
	success rate (%)	(AFRs in FPocket, %)
STINGAllo	60.2	77.8
PASSer Ensemble	21.1	47.4
PASSer AutoML	23.2	45.0
PASSer Rank	24.2	55.0

As shown in Table 2, STINGAllo achieved an overall DCC success rate of 60.2%, outperforming the three different models available through PASSer (Ensemble, AutoML, and Rank), which had success rates ranging from 21.1% to 24.2%. The performance gap was particularly pronounced in the most challenging cases where AFRs were located on flat surfaces or were otherwise missed by pocket-finding algorithms. In the subset of test cases where all known AFRs were correctly located within an FPocket-delineated pocket, STINGAllo’s success rate rose to 77.8%, again substantially higher than the best-performing PASSer model (55.0%). These quantitative results validate the effectiveness of STINGAllo’s residue-centric, IPN-based approach, and underscore its advantages over methods reliant on preliminary pocket detection.

To provide an overview of the current landscape of allosteric site prediction methods, Table 3 presents a comparison of STINGAllo with representative contemporary tools, highlighting the methodological innovations and unique advantages of each approach. The comparison includes PASSer [5, 13, 5], Allosite [3], AlloPred [1], MEF-AlloSite [16], and DeepAllo [17].

**Table 3:** Comparison of STINGAllo with representative allosteric site prediction tools, summarising core features, methodological approaches, and key advantages.

Method	Approach	Descriptor type	Key innovation	Availability	Limitations
**STINGAllo** [12]	Residue-centric ML	IPN descriptors	Pocket-independent prediction	Web server + API	Static structure
PASSer [5, 13]	Pocket-based ML	Geometric + physicochemical	Ensemble learning	Web server + API	Pocket-dependent + Static structure
Allosite [3]	Pocket-based	Geometric descriptors	Cavity scoring	Web server	Pocket-dependent + Static structure
AlloPred [1]	Pocket-based ML	Normal mode analysis	Dynamics integration	Web server	Pocket-dependent + Computationally more expensive
MEF-AlloSite [16]	Multimodel ensemble	Diverse feature sets	Robust feature selection	Standalone tool	Pocket-dependent + Static structure + Limited training data
DeepAllo [17]	Deep learning	Protein language models	pLM + FPocket features	Research prototype	Pocket-dependent + Black box predictions

IPN, internal protein nanoenvironment; ML, machine learning; API, application programming interface; pLM, protein language model.

### Case Study 1: hemoglobin—a classical allosteric protein

Hemoglobin is the quintessential example of allostery in textbooks. This tetrameric blood protein ($\alpha _{2}\beta _{2}$) binds oxygen cooperatively: binding of O$_{2}$ to one subunit increases the affinity of remaining subunits, an effect explained by allosteric transition between low-affinity (T) and high-affinity (R) states (the classic Monod–Wyman–Changeux model). Hemoglobin also has heterotropic allosteric regulators; notably, 2,3-BPG binds to a pocket at the interface of the $\beta $-subunits, stabilizing the T state and thus reducing oxygen affinity. This 2,3-BPG binding pocket is an allosteric site $\sim $35 Å away from the heme iron atoms. Any credible allosteric site predictor should be able to identify this site on hemoglobin, given its prominence and distinct pocket features [18, 19].

We used the STINGAllo web server to analyze human hemoglobin. Specifically, we input PDB 1A01 that is a high-resolution crystal structure of human hemoglobin in the deoxy (T) state. The structure contains four chains (two $\alpha $ and two $\beta $). We focused on the $\beta $ chain (chain A in this PDB) for demonstration, since the 2,3-BPG site is mainly located between the two $\beta $ chains. After submitting the job (Fig. 3C shows the input interface for 1A01 chain A), STINGAllo returned a set of predicted AFRs on the $\beta $ chain. The key residues identified were $\beta $Phe$_{36}$, $\beta $Val$_{96}$, $\beta $Leu$_{100}$, $\beta $Leu$_{101}$, $\beta $His$_{103}$, and $\beta $Asp$_{126}$ (using the $\beta $ chain numbering from PDB 1A01). These residues are highlighted in dark green on the hemoglobin structure in Fig. 3C. Residues such as Val96 and His103 lie at the interface region, suggesting they help propagate the allosteric signal between subunits. Asp126, which sits nearer the cavity entrance, may support ionic or hydrogen-bond interactions that contribute to pocket stability in the T state. Although not all of these positions directly contact 2,3-BPG in canonical structures, they likely participate in the network of residue interactions underlying hemoglobin’s cooperative oxygen-binding mechanism.

### Case Study 2: lac repressor (PDB ID:1EFA, chain A), inducer-binding pocket

The *Escherichia coli* lac repressor (LacI) is a classic allosteric protein in gene regulation. LacI binds DNA to repress transcription, and binding of an inducer (e.g. isopropyl $\beta $-D-1-thiogalactopyranoside (IPTG) or analogs) at a remote site triggers a conformational change that lowers DNA affinity. Crystallographic studies of LacI have captured the protein in both DNA-bound and inducer-bound states, providing a model for how inducer binding at the core domain causes an allosteric transition. The inducer-binding pocket is located in the large core (or C-subdomain) of LacI, between the N- and C-subdomains of the dimeric protein [20]. This pocket is $\sim $20–25 Å away from the DNA-binding domains, and its occupancy by an effector leads to a reorientation of those DNA-binding domains. In the 1EFA structure, LacI is bound to an operator DNA and the anti-inducer orthonitrophenylfucoside (ONPF) in its core pocket [21].

Structural and simulation studies have identified key residues lining this allosteric pocket. For example, Asp149 in the core domain directly contacts the inducer (IPTG in simulations) and lies at the base of a flexible loop, acting as a hinge that propagates the conformational signal [20]. Likewise, Phe161 at the rear of the pocket forms hydrophobic contacts with the inducer, and His74 at the dimer interface forms a pi-stacking interaction that changes upon inducer binding. This extensive network of interactions between the core (inducer-binding) domain and the DNA-binding domain underlies the mechanism of allostery in LacI [21].

In chain A of 1EFA, the STINGAllo-highlighted residues include those surrounding the ONPF ligand (analogous to IPTG), for instance, residues in the loop harboring Asp149 and neighboring hydrophobic residues such as Phe161 (core subdomain)—which are crucial for inducer binding and the ensuing conformational change. These predicted AFRs coincide with experimentally validated allosteric hotspots: Asp149 and its loop act as a “trigger” region that senses ligand binding, while nearby residues form a hydrophobic cluster that transmits the effect toward the interface and DNA-binding domain [22, 23]. The strong agreement between STINGAllo predictions and the inducer pocket is not surprising, this pocket presents a distinct internal protein nanoenvironment (a concave binding site with specific polar contacts and hydrophobic patches) that differs from typical solvent-exposed surfaces. STINGAllo’s per-residue analysis likely detects features such as the network centrality of these residues (since they lie at a junction between domains) and their moderate distance from the protein’s center, both of which are characteristic of known allosteric sites in LacI.

**Figure 4:**
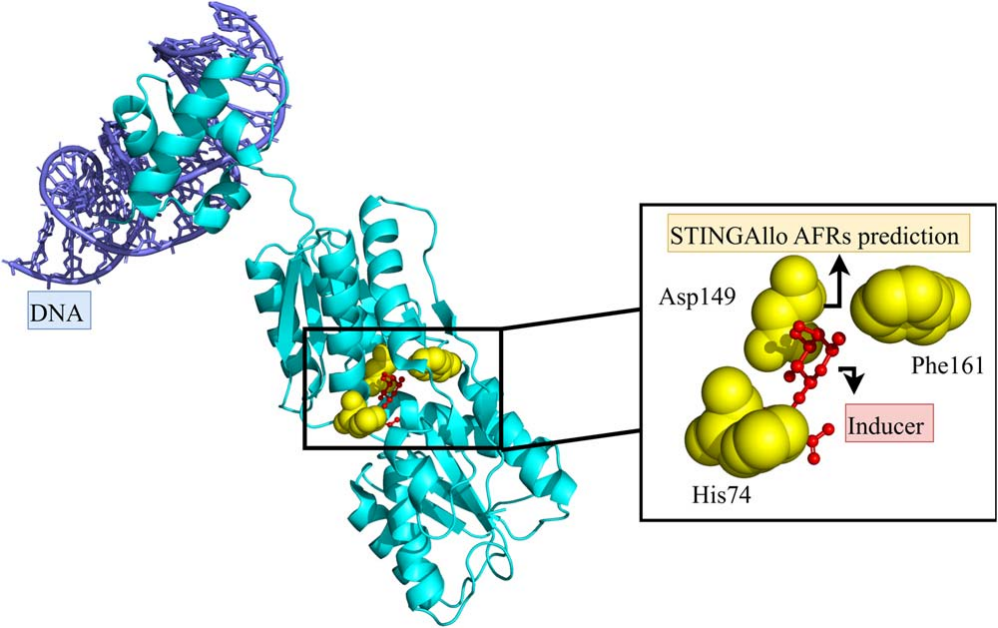
Structure of the *E. coli* lac repressor (PDB: 1EFA, chain A) showing STINGAllo-predicted AFRs within the inducer-binding pocket in complex with operator DNA, illustrating agreement with experimentally validated allosteric hotspots.

The predicted AFRs in LacI cluster at the interdomain junction where inducer binding triggers reorientation. Structurally, this makes them strong allosteric candidates because they occupy a communication hub: they are located in the core domain pocket (the site of ligand binding) but directly interact with elements (loops and helices) that connect to the DNA-binding domain and the dimer interface. This explains why mutations in this region or ligand binding have outsized effects on function. In LacI’s induced state, the STINGAllo-identified residues engage in altered interactions (e.g. the breakage of a Lys84–Lys84’ inter-subunit contact and a shift in helix positioning [20, 21]) that relieve repression (Fig. 4).

### Case Study 3: glutamate racemase (PDB ID:2W4I, chains A/B)—dimer-interface pocket

**Figure 5:**
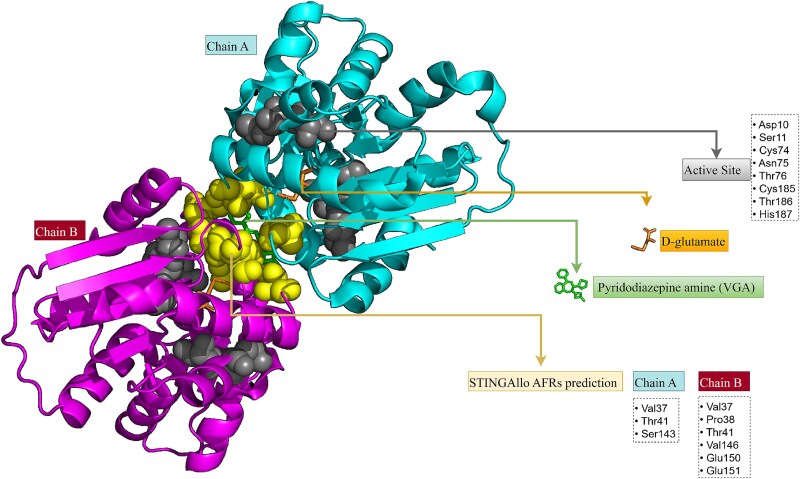
Structure of *H. pylori* glutamate racemase (PDB: 2W4I) showing the dimer-interface allosteric pocket with STINGAllo-predicted AFRs overlaid on experimentally determined residues, highlighting strong agreement between predictions and known functional sites.

*Helicobacter pylori* glutamate racemase (MurI) is a homodimeric enzyme essential for peptidoglycan biosynthesis, converting L-glutamate to D-glutamate. Unlike many enzymes that are regulated by feedback metabolites, MurI lacks obvious endogenous allosteric regulators, however, drug discovery efforts have identified small-molecule inhibitors that bind outside the active site [24] (Fig. 5). In the 2W4I structure, MurI is co-crystallized with its substrate (D-glutamate) bound in the active site and a synthetic inhibitor bound at an unexpected site, the dimer interface [25]. This inhibitor is a pyridodiazepine amine (originating from a high-throughput screen hit optimized via structure–activity relationship) and was shown to act as an uncompetitive inhibitor, preferentially binding the enzyme–substrate complex [25]. The literature thus defines a novel allosteric pocket at the interface of the two MurI monomers: the inhibitor lodges between chain A and chain B, $\sim $5 Å away from the catalytic residues, and “braces” the dimer in a way that likely hinders the conformational dynamics required for catalysis. The binding site spans both subunits, notably involving interface loops and helices that are distant from the active-site clefts in each monomer. This dimer-interface allosteric site is evident from the crystal structure and biochemical data: the inhibitor does not overlap the racemase’s orthosteric (substrate-binding) site, which is consistent with allosteric inhibition. Geng *et al.* [25] explicitly noted that these inhibitors “bind at the MurI dimer interface” rather than in the active pocket as shown in Fig. 5.

The STINGAllo prediction for 2W4I highlights a set of residues located at the homodimer interface that match the experimentally determined allosteric pocket (Fig. 5). Specifically, STINGAllo flags residues from both chain A and chain B that form the inhibitor-binding (STINGAllo was run separately on each chain, and the results were subsequently integrated to yield the dimer interface). For example, interface residues from chain A’s $\beta $-sheet and the opposing loop from chain B (which together cradle the inhibitor) are predicted as AFRs. These include side chains that directly contact the bound inhibitor (pyridodiazepine), such as a conserved aromatic or hydrophobic residue that the inhibitor stacks against, and polar residues that form hydrogen bonds with the inhibitor. Notably, STINGAllo does not erroneously flag the active-site residues as allosteric; rather, it focuses on this remote site, reflecting its ability to distinguish the unique internal protein nanoenvironment of an allosteric pocket from that of the orthosteric site. The predicted interface residues coincide with known important sites: for instance, tyrosine or phenylalanine residues at the dimer interface, if they form part of the inhibitor pocket, likely appear in STINGAllo’s output because their IPN (buried at an interface with unusual “druggable” pocket properties) is characteristic of allosteric hotspots. The congruence between prediction and the known site suggests that the dimer interface pocket possesses distinctive features (e.g. high structural packing density and network centrality connecting the two subunits) that STINGAllo has successfully identified. Disruption of this region is known to impair enzyme function, which validates its allosteric importance.

### Case Study 4: glucosamine-6-phosphate synthase (Gfa1p, PDB ID: 2PUV)—Allosteric feedback inhibition by uridine diphosphate-*N*-acetylglucosamine

**Figure 6:**
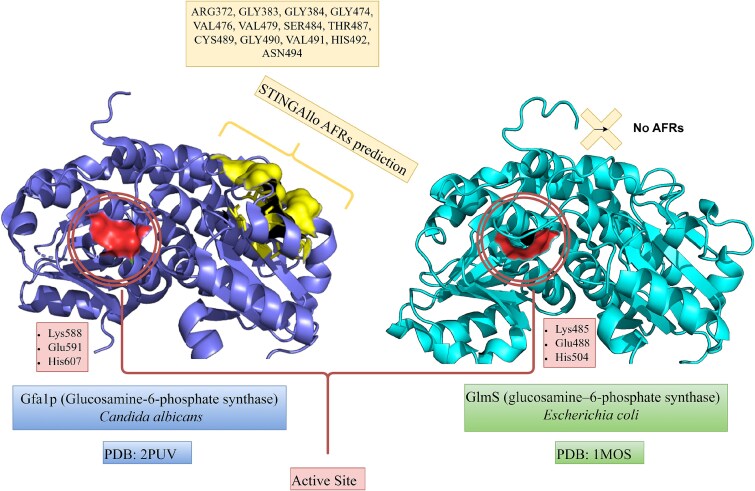
Structure of glucosamine-6-phosphate synthase (Gfa1p, PDB: 2PUV) showing STINGAllo-predicted AFRs in the C-terminal isomerase domain at the allosteric feedback inhibition site bound by UDP-N-acetylglucosamine, illustrating agreement with experimentally characterised regulatory residues.

STINGAllo predicted AFRs (chain A) are Arg372, Gly383, Gly384, Gly474, Val476, Val479, Ser484, Thr487, Cys489, Gly490, Val491, His492, and Asn494 shown as yellow surface in Fig. 6. These residues lie in the C-terminal isomerase domain of Gfa1p, and many cluster around a specific surface pocket and oligomeric interface region. This suggests that STINGAllo has pinpointed the allosteric inhibitor-binding pocket and adjacent structural elements that mediate feedback control.

Glucosamine-6-phosphate synthase catalyzes the first committed step in uridine diphosphate-*N*-acetylglucosamine (UDP-GlcNAc) production, a precursor required for fungal cell wall components (e.g. chitin and other glycans) [26]. In eukaryotes (such as fungi), the enzyme (known as Gfa1p in yeast) is subject to feedback inhibition by UDP-GlcNAc, the end-product of its pathway. By contrast, STINGAllo identifies no AFRs in the bacterial homolog GlmS [27], consistent with its lack of allosteric regulation, thereby highlighting the eukaryote-specific evolutionary addition of a regulatory site in Gfa1p (Fig. 6). This feedback mechanism is crucial for metabolic balance: Gfa1p is essential for cell wall biosynthesis in fungi and is a target for antifungal therapy [26]. The enzyme is a homotetramer in eukaryotes, and each subunit consists of two domains: an N-terminal glutamine amidotransferase domain and a C-terminal isomerase domain where fructose-6-phosphate is converted to glucosamine-6-phosphate.

Structural studies have provided insight into how UDP-GlcNAc inhibits Gfa1p. Crystal structures of *Candida albicans* Gfa1p (isomerase domain) in complex with UDP-GlcNAc show the inhibitor bound to a distinct pocket on the surface of the isomerase domain, $\sim $10 Å away from the active site [26, 28]. The allosteric inhibitor binds alongside a coordinated metal cation in this pocket. Notably, the residues that form the UDP-GlcNAc binding pocket (and interact with the inhibitor) are conserved only in eukaryotic sequences of GlcN6P synthase. This corresponds well with the STINGAllo predictions, which identified a cluster of residues as shown in Fig. 6. Many of these residues are part of the eukaryote-specific regulatory pocket that does not exist in the bacterial enzyme, explaining why only the eukaryotic enzyme is allosterically regulated.

Binding of UDP-GlcNAc to this remote site induces long-range effects on the enzyme’s conformation and dynamics. While the static crystal structure captures the inhibitor nestled in its pocket, solution studies and MD have shown that the ligand-bound enzyme becomes more rigid, especially in regions critical for catalysis [28]. In the absence of UDP-GlcNAc, the isomerase domain and connected regions exhibit greater flexibility (necessary for the enzyme’s catalytic cycle), but when UDP-GlcNAc is bound, it “locks” the enzyme in a more fixed, less active conformation. In essence, UDP-GlcNAc acts as an allosteric brake: it binds away from the active site [26, 29] and stabilizes an inhibited state of Gfa1p, thereby slowing the production of its own precursor. This is a classic feedback inhibition loop that prevents over-accumulation of UDP-GlcNAc and is explored here using STINGAllo.

## Discussion

STINGAllo represents a significant advancement in allosteric site prediction by offering per-residue resolution, which is crucial for understanding the molecular determinants of allosteric regulation. Our previous publication introduced the STINGAllo model and demonstrated its performance in a controlled benchmark setting [12]. However, without an accessible interface, that model might remain underutilized by experimentalists or even by computational biologists who are not experts in machine learning. By developing the web server and API, we have effectively translated a computational model into an interactive tool that anyone can use. Researchers can now leverage STINGAllo predictions in planning experiments, whether it is deciding which mutations to make to test allosteric regulation or identifying new pockets for small-molecule binding, in a matter of minutes. The case studies presented in the Results section exemplify this capability: an enzymologist studying ATCase can quickly verify that STINGAllo identifies the adenosine triphosphate/cytidine triphosphate (CTP) regulatory site, while a microbiologist working on a newly solved protein structure can obtain clues about possible regulation without resorting to lengthy simulations.

The inclusion of a RESTful API is a forward-looking feature that we expect will encourage the integration of STINGAllo into larger workflows. For example, a medicinal chemist using a pipeline to screen for druggable sites across a pathogen’s proteome can integrate STINGAllo’s API calls after structure prediction to highlight allosteric pockets. Similarly, databases of allosteric sites could incorporate STINGAllo predictions as a supplemental layer, perhaps flagging proteins that harbor additional predicted sites beyond those already known. The interoperability offered by the API means that STINGAllo’s utility can extend far beyond its own interface, potentially being called by other web servers or tools. Such cross-platform synergy would greatly amplify the impact of allosteric site prediction in everyday research.

STINGAllo is not a solitary solution but part of an ecosystem of complementary approaches [12]. Each method, whether based on static pocket analysis, dynamics, or network analysis, sheds light on allostery from different perspectives. As our quantitative comparison demonstrates, STINGAllo offers a distinct advantage in its ability to identify sites that are not dependent on pre-defined pockets, complementing tools that excel in pocket analysis. We foresee that ensemble approaches, which combine the outputs of multiple predictors, might become popular to achieve higher confidence. In such a scenario, STINGAllo’s predictions would serve as one vote in a consensus method. For instance, given its demonstrated ability to capture subtle signals, STINGAllo might function as an “early warning” system for potential allostery even when other pocket finders reveal nothing. Conversely, if a pocket finder highlights a site that STINGAllo does not, this might indicate a pocket, i.e. functionally inert or one that lies outside STINGAllo’s training sphere (e.g. a membrane-embedded allosteric site). In short, STINGAllo should be viewed as complementary to existing tools, with combined use strengthening the overall conclusions.

From a methodological perspective, the success of STINGAllo reinforces the notion that rich structural descriptors and machine learning can capture subtle signals of allostery. This echoes findings from related work, which have shown that AFRs often possess distinguishing properties or unique physicochemical properties. The descriptors deemed important by STINGAllo (e.g. the sponge effect and graph bottlenecks) [12] may inspire further research into the biophysical underpinnings of allostery and prompt new experiments comparing known allosteric and non-allosteric sites.

## Limitations, mitigation strategies, and future directions

While STINGAllo demonstrates strong predictive performance, it is essential to acknowledge its limitations and outline strategies for future improvement.

### Predictive precision and false positives

A primary limitation is the potential for false positives and negatives. The definition of an allosteric “site” can be ambiguous, spanning from direct ligand-binding residues to a wider network involved in conformational transitions. STINGAllo is trained to identify AFRs, but its predictions can sometimes encompass this broader communication network. A clear illustration is the hemoglobin case study (PDB 1A01). When benchmarked only against the classical 2,3-BPG cavity ($\beta $ Val1, $\beta $ His2, $\beta $ Lys82, and $\beta $ His143) [30, 31], STINGAllo seems to yield false positives: none of its six $\alpha $-chain AFRs or two $\beta $-chain AFRs contact the effector directly. Instead, the algorithm highlights Phe36, Val96, Leu100–101, His103, and Asp126 on the $\alpha $-chain and Phe103/Leu105 on the $\beta $-chain (Fig. 3).

Structural and mutational studies show that these residues cluster at the $\alpha _{1}\beta _{2}$ interface, the “switch” and “hinge” that must shear and repack during the T $\rightarrow $ R quaternary transition [32–34]. Thus, while the site-level precision for the BPG pocket is low, the model accurately pinpoints a high-impact allosteric region, underscoring a common evaluation pitfall: success in mapping the allosteric pathway can masquerade as failure when the gold-standard set is limited to ligand-contact residues.

Similar apparent discrepancies are expected whenever a protein’s regulation is mediated by long-range conformational shifts (e.g. kinases with activation loops or GPCRs with microswitch networks) that lie outside a well-defined small-molecule pocket.

### Biases in training data and model applicability

The performance of STINGAllo is contingent on its training data (ASBench), which, despite being a standard benchmark, has inherent biases. The dataset is dominated by globular enzymes and small soluble signaling proteins, accounting for the vast majority of ASBench entries, while membrane-embedded, multimeric, or intrinsically disordered region (IDR)-rich systems are largely absent [35]. A significant, known limitation is the model’s unvalidated performance on membrane proteins and IDR-rich systems. Membrane proteins function within a lipid bilayer, a unique physicochemical environment not explicitly captured by our training set. Consequently, applying STINGAllo to transmembrane proteins like GPCRs or ion channels is not recommended without further validation. Current studies are ongoing to address this limitation, which requires a concerted effort to expand the training set with curated examples of allosteric membrane proteins.

Similarly, the model may be less accurate for very large macromolecular complexes, proteins where allostery is mediated by IDRs, or mechanisms driven by covalent modification. The current server’s one-chain-at-a-time processing is also a usability limitation for large complexes.

*Mitigation and future directions.* To broaden the scope of *STINGAllo*, we will implement four complementary upgrades. First, we will augment the current *ASBench*-derived training set with experimentally annotated IDRs from DisProt v8.0, thereby enabling the model to recognize allosteric communication mediated by flexible segments that are virtually absent from the existing benchmark [36]. Second, we will curate a “STING–Membrane” subset from *ASD 2023*, which now documents dozens of G-protein-coupled receptors, ion channels, and transporters with validated allosteric modulators, providing the necessary lipid-embedded exemplars for robust membrane-protein prediction [37]. Third, inspired by recent successes of MD-augmented allostery predictors [38–41], we will pre-compute short (100 ns) equilibrium trajectories for all training structures and distil them into lightweight per-residue ensemble descriptors, such as RMSF, hydrogen-bond persistence, and contact lifetimes, that can be consumed at inference without obliging users to run MD. Finally, we will introduce a batch-submission API for high-throughput analysis and upgrade the web viewer to overlay AFRs scores with catalytic residues and ligand contacts in an interactive split-screen, a design shown to facilitate hypothesis generation [42]. Collectively, these extensions will extend *STINGAllo* to membrane proteins, IDR-rich regulators, and large assemblies, while delivering faster, richer feedback to both researchers and students.

The platform is freely available at https://www.stingallo.cbi.cnptia.embrapa.br, and we invite the community to explore its capabilities, provide feedback, and share both success stories and challenges.

## Conclusion

Allostery is a pervasive phenomenon in biology that offers attractive opportunities for therapeutic intervention due to its potential for high specificity and modulatory control [12]. Although computational prediction of allosteric sites has been challenging, recent advances in machine learning and structural bioinformatics have markedly improved our capabilities. In this work, we presented the STINGAllo Web Server and API as a user-friendly platform for allosteric site prediction, building on our previously published STINGAllo model. We highlight the intuitive user interface, technical innovations such as RESTful API integration and validation through multiple case studies. By making allosteric site predictions accessible to a broad range of researchers, STINGAllo paves the way for novel discoveries in protein function and drug design, ultimately contributing to the expansion of the druggable genome.

**Key Points**

- STINGAllo predicts allosteric site-forming residues at the per-residue level using machine learning and internal protein nanoenvironment descriptors, achieving a 78% accuracy on benchmark datasets.
- The tool processes over 227 000 Protein Data Bank structures, identifying allosteric potential in 52.7% of analyzed proteins.
- It integrates interactive 3D visualization and a RESTful application programming interface, facilitating accessibility for computational and experimental researchers.
- STINGAllo outperforms existing pocket-based prediction methods by offering fine-grained, residue-specific insights crucial for mutagenesis, and drug design studies.
- The platform supports local file uploads, enabling researchers to analyze newly determined structures immediately, and assess potential allosteric sites without waiting for database updates.

## Supplementary Material

Briefings_Revision_Supplementary_30th_June_bbaf424

## Data Availability

The datasets generated and analyzed during this study, including the STINGAllo predictions and underlying descriptor data, are available from the corresponding author upon reasonable request. The STINGAllo web server is publicly accessible at https://www.stingallo.cbi.cnptia.embrapa.br/, and the API documentation is available at https://www.stingallo.cbi.cnptia.embrapa.br/apis. All datasets used for training the STINGAllo model (e.g. ASBench) are publicly available, as referenced in our previous publication [12].

## Abbreviations

- AFRs: allosteric site-forming residues
- FR: free residues
- LASA: loss of accessible surface area
- PDB: Protein Data Bank
- API: application programming interface
- DCC: distance to center
- ROC-AUC: receiver operating characteristic-area under the curve
- MCC: Matthews correlation coefficient
- IPN: internal protein nanoenvironment
- MD: molecular dynamics
- MSSP: multiple structure single parameter
- GTP: guanosine triphosphate
- BPG: bisphosphoglycerate
- UDP-GlcNAc: uridine diphosphate $N$-acetylglucosamine
- SAR: structure–activity relationship
- cAMP: cyclic adenosine monophosphate
- ATP: adenosine triphosphate
- NMR: nuclear magnetic resonance
- HTS: high-throughput screening
- RCSB: Research Collaboratory for Structural Bioinformatics
- CNPTIA: Embrapa’s National Center for Research in Information Technology in Agriculture
