# Supplementary material for: STINGAllo: a web server for high-throughput prediction of allosteric site-forming residues using internal protein nanoenvironment descriptors
Source: Brief Bioinform. 2025 Aug 21;26(4):bbaf424. doi: 10.1093/bib/bbaf424 (PMC12368853; doi:10.1093/bib/bbaf424)
Supplement: Briefings_Revision_Supplementary_30th_June_bbaf424 [file briefings_revision_supplementary_30th_june_bbaf424.pdf]

Journal Title Here, 2025, pp. 1–5

doi: DOI HERE
Advance Access Publication Date: Day Month Year
Paper

PAPER

Supplementary

Folorunsho Bright Omage,[1,2,†] José Augusto Salim,[3] Ivan Mazoni,[1] Inácio Henrique Yano,[1] Jorge Enrique Hernández González,[4] Poliana Fernanda Giachetto,[1] Ljubica Tasic,[2] Raghuvir Krishnaswamy Arni[4] and Goran Neshich[1,†]

[1]Computational Biology Research Group, Embrapa Digital Agriculture, Campinas, , São Paulo, Brazil, [2]Biological Chemistry Laboratory, Department of Organic Chemistry, Institute of Chemistry, University of Campinas (UNICAMP), Campinas, , São Paulo, Brazil, [3]Department of Plant Biology, Institute of Biology, University of Campinas (UNICAMP), Campinas, , São Paulo, Brazil and [4]Institute of Biosciences, Humanities and Exact Sciences, São Paulo State University (UNESP), São José do Rio Preto, Brazil

[†]Corresponding author: Folorunsho Bright Omage. omagefolorunsho@gmail.com[†]Corresponding author: Goran Neshich. g.neshich@embrapa.br

FOR PUBLISHER ONLY Received on Date Month Year; revised on Date Month Year; accepted on Date Month Year

Abstract

Materials and methods

Backend Development

STINGAllo is implemented as a Flask-based web application with a relational database backend for managing structural descriptors and prediction results. The core prediction engine employs a CatBoost gradient-boosted decision tree model, selected for its robustness in handling complex interactions within high-dimensional datasets. The model was trained on a curated dataset of 1,200+ structural and physicochemical descriptors derived from the STING database. These descriptors capture key protein features, including solvent accessibility, electrostatic potential, hydrophobic interactions, and graph-theoretical connectivity[1].

STINGAllo accepts protein structure input in two forms: (1) a PDB identifier, or (2) a user-uploaded PDB format file. For a PDB ID query, the backend first validates the identifier format (a four-character alphanumeric code) and allows the user to select the chain. It then retrieves the corresponding AFRs for that PDB ID. All data transfers use secure connections (HTTPS) and the retrieved file is temporarily cached on the server. For uploaded files, the server verifies that the file is indeed in PDB format (text with ATOM/HETATM records). A parsing module reads the coordinate data from the source. During parsing, the server extracts relevant metadata (chain IDs, sequence, etc.) and if a structure contains multiple chains, the user is prompted to specify a chain.

A key advantage of STINGAllo is its use of rich internal protein nanoenvironment descriptors from the STING database, which were already successfully used in previous studies to fully characterize some of the most studied internal protein nanoenvironments[2, 3, 4, 5, 6, 7, 8, 9]. A comprehensive set of features is gathered for each residue in the input structure to characterize its IPN. These features include solvent accessibility, secondary structure context, hydrophobic interaction energy, electrostatic potential, proximity to the protein's center of mass, graph-theoretic metrics (e.g., network centrality or "bottleneck" scores), sponge effect indicating local packing density, and other biochemical descriptors.

Once the input structure is parsed and its features are ready, the server executes a step-by-step prediction pipeline to classify allosteric residues. For each amino acid residue in the structure, the system obtains the full set of selected descriptors, invoking routines for surface area calculation, distance measurements, and other pre-defined STING descriptors, as detailed in our previous articles[1, 7]. The result is a feature matrix where each row corresponds to one residue and each column to a particular descriptor value. The feature matrix is then fed into the CatBoost classification models. STINGAllo routes each residue's feature vector to the appropriate CatBoost model that was trained for that residue's amino acid type. In practice, this is handled seamlessly, the system can batch-process all residues by grouping them by amino acid and running predictions for each group with the corresponding model. The CatBoost models output 1 for the residue being an AFR.

The server collates the per-residue predictions into a structured result. This includes the residue identifier (chain ID and residue number) and residue type. The data are then formatted as a table for the web page and as a JSON object for API responses. For the web interface, an HTML results page is generated, including an interactive 3D viewer that highlights the predicted allosteric residues on the protein structure (This feature can be toggled on or off). For API requests, the server returns a JSON response containing the results. This entire pipeline executes in an automated fashion for each submission, typically completing within a few seconds,

© The Author 2025. Published by Oxford University Press. All rights reserved. For permissions, please e-mail: journals.permissions@oup.com

thereby providing near-real-time feedback to the user. The computational efficiency of STINGAllo is a crucial factor in ensuring real-time application feasibility. As a web server designed to handle multiple simultaneous requests, it process large datasets efficiently while maintaining rapid response times. This is achieved through parallelized batch processing and a multi-worker architecture, which collectively minimize latency and maximize throughput.

Optimization for Speed and Scalability

To ensure efficient processing, STINGAllo employs a multi-threaded pipeline for feature extraction and parallelized prediction execution. Descriptor calculations are optimized via batch processing, and Gunicorn workers handle concurrent API requests. Server-side caching mechanisms accelerate retrieval of previously computed predictions, reducing response times for commonly queried structures.

In terms of request handling, the architecture is designed to be stateless, enabling horizontal scaling if needed. Each HTTP request carries all the information needed for AFRs retrieval, and the server can be replicated behind a load balancer to distribute incoming jobs. We have also implemented timeouts and resource limits to prevent excessively large requests from monopolizing the server, majorly on API requests. For instance, too many API requests from a single user will be detected and rejected with a message to maintain quality of service.

Deployment and Infrastructure

The STINGAllo server is deployed on a Linux-based environment configured for high availability and security. The application is hosted behind an NGINX reverse proxy, which serves static content (CSS/JS for the web interface) and forwards API calls to the backend application server. We use Gunicorn (a WSGI application server) to run the Python app with multiple worker processes, enabling the handling of concurrent requests. The server is configured to use HTTPS exclusively, employing TLS encryption to secure data in transit, this is particularly important for protecting user-uploaded structures and ensuring integrity of data exchange. User uploads are handled in a secure sandbox directory on the server; after processing, files are automatically deleted to avoid accumulation of sensitive data on the disk. The underlying relational database (`STING_RDB`) is hosted on a dedicated database server (MySQL), and connections from the web application to the database are managed via secure credentials and limited privileges (read-only access for descriptor retrieval, for example).

Security considerations are integral to the deployment. Only PDB-formatted content is accepted, and the upload interface strictly checks file type and size to prevent any malicious file upload. The web framework's built-in protections (against SQL injection, cross-site scripting, etc.) are enabled and kept up-to-date. We also implemented rate limiting at the NGINX layer to mitigate denial-of-service attacks, if an IP address makes an unusually high number of requests in a short time, subsequent requests may be delayed or blocked. Regular backups of the database and model files are performed, which simplifies deployment and provides an additional layer of isolation for the running process.

Methodological Considerations and Rationale for Case Study Selection

We chose a wide variety of protein systems with the intent to explore and demonstrate the potential of STINGAllo to predict AFRs. Importantly, none of the proteins featured in these case studies were included in our machine learning model's training set, making the predictions nontrivial. Two main considerations guided our selection strategy. To validate and demonstrate STINGAllo performance by recapitulating well-known allosteric systems. Classical models like hemoglobin and the lac repressor have provided important benchmarks for allostery, with their mechanisms of regulation being quantitatively characterized. Second, we sought to broaden the applicability of STINGAllo to more elaborate regulatory contexts—covering not just protein monomers but also protein dimers, multi-domain enzymes, cryptic allosteric sites, and allosteric feedback inhibition systems.

Results

Case Study 4: cAMP-Activated Acetyltransferase (Pat; Rv0998) — PDB 4AVC: Cryptic cAMP-Mediated Allosteric Activation

STINGAllo Predicted Allosteric Residues for (Chain A) are: Val67, Arg80, Ala81, Gly88, Glu89, Arg98, Ser99, Arg138, Ala141, Phe142. These predicted AFRs are in the protein's N-terminal regulatory domain and at the interface with the C-terminal acetyltransferase domain. Notably, several predicted residues (e.g., Glu89, Arg98, Arg138, Phe142) correspond to the cyclic AMP (cAMP) binding pocket and the interdomain "latch" region that controls activation.

Mycobacterium tuberculosis Pat (also called Mt-PatA) is a Gcn5-related lysine acetyltransferase fused to a cyclic nucleotide–binding domain, which enables direct regulation by cAMP. Crystal structures of Pat in its autoinhibited and cAMP-bound forms reveal that cAMP binds to a cryptic site in the regulatory domain, approximately 32 Å away from the catalytic acetylation site. cAMP binding triggers a large conformational rearrangement that relieves autoinhibition by displacing a "substrate-mimicking" lid that normally blocks the substrate binding surface. A steric double latch mechanism couples the regulatory and catalytic domains: in the absence of cAMP, the protein's C-terminal tail occupies the cAMP pocket and, together with the lid, keeps the enzyme inactive[10]. Upon cAMP binding, this latch is released, the C-terminal tail is ejected from the pocket and the lid opens, allowing the catalytic site to access substrate.

The STINGAllo-predicted residues align well with known allosteric sites. For example, cAMP binds at the N-terminus of a regulatory helix contacting Glu89 and Arg98, while Arg138 and Phe142 (in a helix termed "Helix F") reorient to interact with cAMP's adenine ring[10]. These residues have been identified as critical for the cAMP-mediated conformational switch, since mutating the conserved cAMP-contacting residues to alanine abolishes allosteric activation. Thus, the predicted allosteric residues correspond to the key cAMP-binding pocket and latch interface that drive activation. Experimentally, cAMP-mediated domain rotation and lid refolding have been observed, confirming that Pat undergoes a dramatic rearrangement upon effector binding. This cryptic allosteric regulation illustrates how Pat directly links a second messenger (cAMP) to enzymatic

activity, a novel mechanism for *M. tuberculosis* acetylation control[10].

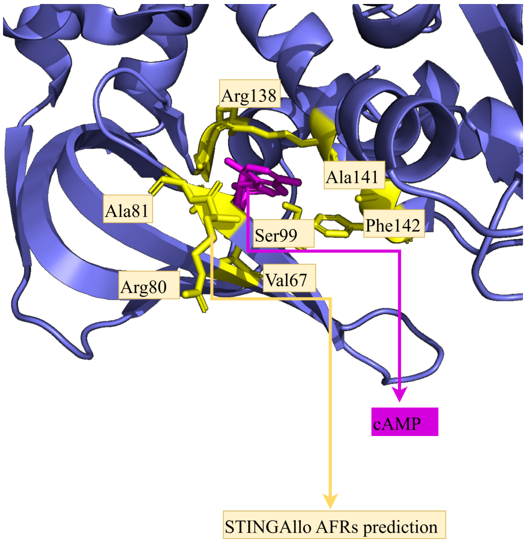

Fig. 1. cAMP-Activated Acetyltransferase (Pat; Rv0998) – PDB 4AVC: Cryptic cAMP-Mediated Allosteric Activation. The structure of *Mycobacterium tuberculosis* Pat (PDB 4AVC, chain A) is shown in a blue cartoon representation with the STINGAllo-predicted allosteric residues highlighted in yellow (displayed as sticks). These residues (Val67, Arg80, Ala81, Gly88, Glu89, Arg98, Ser99, Arg138, Ala141, and Phe142) are clustered in the N-terminal regulatory (cyclic nucleotide-binding) domain and at the interface with the C-terminal acetyltransferase domain. The cAMP ligand, depicted in a magenta stick, is bound to the cryptic pocket.

Case Study 5: Pyruvate Kinase (LmPYK) – PDB 3HQP: Allosteric Activation by Fructose 2,6-Bisphosphate in Parasite Glycolysis

STINGAllo Predicted Allosteric Residues (Chain A) are Leu399, Ser400, Asn401, Thr402, Arg404, Ser405, Lys453, Arg456, His480, Ala481, Val485, Lys486, Gly487, Tyr488, Ala489. These predicted residues form two notable clusters: one around the interfaces of the subunits/domains (399–405 region) and another in the effector-binding region (453–489 loop segment). This suggests that STINGAllo has identified the regions involved in the allosteric transition and effector binding of pyruvate kinase.

Leishmania mexicana pyruvate kinase (LmPYK) is a key glycolytic enzyme that is unusually regulated by fructose 2,6-bisphosphate (F2,6BP) as an allosteric activator (in contrast to most organisms, which use fructose 1,6-bisphosphate). F2,6BP binding induces a shift from the low-activity T-state to the high-activity R-state, accompanied by substantial quaternary structural changes. X-ray crystallography and comparisons with other pyruvate kinases show that the allosteric transition involves significant rearrangements: a tightly packed array of helices at the subunit interface near the active site is reconfigured, and a ten-stranded inter-subunit β-sheet adjacent to the effector site undergoes bending in the R-state[11]. These conformational changes tighten the relative orientation of the enzyme's domains, promoting the active conformation.

At the F2,6BP effector-binding site, specific structural elements explain the unique activator specificity of LmPYK. Two loops (residues 443–453 and 480–489) adopt markedly different conformations in *Leishmania* versus organisms like yeast, providing the basis for recognizing F2,6BP[11]. Critically, Lys453 and His480, which are present in trypanosomatid pyruvate kinases but not in mammals or yeast, serve as ligands for the 2-phosphate group of F2,6BP. This matches the STINGAllo predictions: Lys453 and His480 were among the top predicted allosteric residues, underscoring their role in binding the allosteric activator. Additional predicted residues in this region (e.g., Arg456, Lys486, Tyr488) likely contribute to stabilizing F2,6BP or to the propagation of its binding signal across the subunit interface. Overall, the predictions coincide with known allosteric hotspots that undergo conformational reorganization upon effector binding.

The allosteric activation of LmPYK by F2,6BP allows the parasite to finely tune its glycolytic flux. Glycolysis is the primary source of ATP in *Leishmania*, and LmPYK serves as a key regulatory point[11]. The use of F2,6BP as an effector is a unique adaptation in trypanosomatid parasites, ensuring pyruvate kinase is optimally active only under conditions when this signal metabolite is present. Importantly, the differences in allosteric sites between parasite and host enzymes offer an opportunity for selective inhibition; the distinctive loops (containing Lys453, His480, etc.) could be targeted by drugs to disrupt the parasite's glycolysis without affecting the host's pyruvate kinase[11]. Thus, integrating STINGAllo predictions with experimental data highlights not only the mechanism of F2,6BP-mediated activation (T-state to R-state transition) but also the potential for parasite-specific therapeutic interventions.

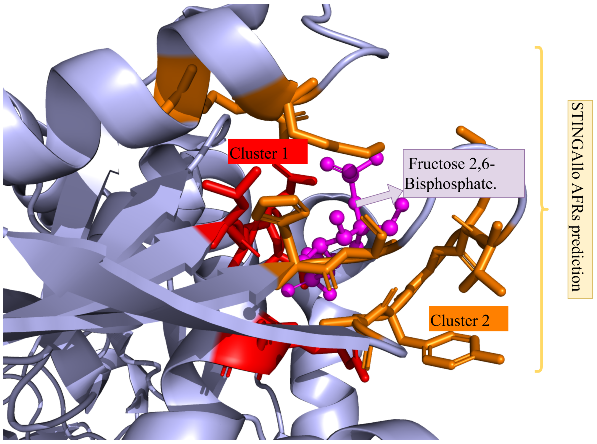

Fig. 2. Allosteric Activation of *Leishmania mexicana* Pyruvate Kinase (LmPYK, PDB 3HQP) by Fructose 2,6-Bisphosphate. The structure of LmPYK (chain A) is shown in cartoon representation (light blue), with STINGAllo-predicted allosteric residues highlighted as sticks. Two distinct clusters of predicted residues are observed: Cluster 1 (red) comprises residues `Leu399, Ser400, Asn401, Thr402, Arg404, and Ser405`, which are located at the interdomain or subunit interface, while Cluster 2 (orange) includes residues `Lys453, Arg456, His480, Ala481, Val485, Lys486, Gly487, Tyr488, and Ala489` in the effector-binding region. These clusters correspond to the regions involved in the conformational transition from the low-activity T-state to the high-activity R-state upon binding of the allosteric activator, fructose 2,6-bisphosphate in a magenta ball and stick representation.

Table 1. A Taxonomy of Computational Methods for Allosteric Site Prediction. This table categorizes representative computational tools and methodologies for predicting allosteric sites on proteins, organized by their core approach.

Category	Representative Tool / Method	Key Principle
**1. Static Pocket-Geometry**	Allosite [12]	Scores surface pockets using geometric and physicochemical features with a Support Vector Machine (SVM).
	Fpocket [13]	Identifies and characterizes protein cavities based on Voronoi tessellation.
	SiteMap [14]	Characterizes binding sites based on occupancy, hydrophobicity, and hydrogen-bonding properties.
	AlloFinder [15]	Combines pocket detection, docking and scoring modules to discover cryptic/allosteric pockets and modulators; accepts user-supplied MD snapshots for conformational diversity.
**2. Dynamics-Driven**		
Normal-Mode Analysis (NMA)	AlloPred [16]	Uses NMA to calculate the dynamic response of a protein to ligand binding perturbations at each pocket.
	PARS [17]	Perturbs the protein structure with a ligand probe and uses NMA to identify coupled "dynamic hotspots."
Molecular Dynamics (MD)	Markov State Models (MSMs) [18]	A statistical framework to model long-timescale dynamics from multiple short MD simulations to identify metastable allosteric states.
**3. Hybrid Machine-Learning**	PASSer [19, 20]	Employs an ensemble of ML models (XGBoost, GCN) trained on structural, energetic, and evolutionary features.
	DeepAllo [21]	Uses a fine-tuned protein-language-model plus pocket descriptors in a deep neural network to predict allosteric sites directly from static structures.
**4. Residue-Centric Network**	RINalyzer / RINs [22]	Models protein structures as Residue Interaction Networks (RINs) to analyze pathways and identify critical nodes (residues) using centrality measures.
	**STINGAllo (This work)** [1]	Classifies individual residues based on a large set of descriptors characterizing the Internal Protein Nanoenvironment (IPN).

References

1. F. B. Omage, J. A. Salim, I. Mazoni, et al. Protein allosteric site identification using machine learning and per amino acid residue reported internal protein nanoenvironment descriptors. *Computational and Structural Biotechnology Journal*, 23:3907–3919, 2024. Published 2024 Oct 23.
2. Goran Neshich, Roberto C. Togawa, Adauto L. Mancini, Paula R. Kuser, Michel E. B. Yamagishi, Georgios Pappas, Wellington V. Torres, Tharsis Fonseca e Campos, Leonardo L. Ferreira, Fabio M. Luna, Adilton G. Oliveira, Ronald T. Miura, Marcus K. Inoue, Luiz G. Horita, Dimas F. de Souza, Fabiana Dominiquini, Alexandre Aĺvaro, Cleber S. Lima, Fabio O. Ogawa, Gabriel B. Gomes, Juliana F. Palandrani, Gabriela F. dos Santos, Esther M. de Freitas, Amanda R. Mattiuz, Ivan C. Costa, Celso L. de Almeida, Savio Souza, Christian Baudet, and Roberto H. Higa. Sting millennium: a web-based suite of programs for comprehensive and simultaneous analysis of protein structure and sequence. *Nucleic Acids Research*, 31(13):3386–3392, July 2003. 1 July 2003.
3. G. Neshich and et al. Sting millennium suite: Integrating network-accessible programs for protein structure analysis. *Nucleic Acids Research*, 33:W94–W98, 2005.
4. Stanley R. M. Oliveira, Gustavo V. Almeida, Kassyus R. R. Souza, Diego N. Rodrigues, Paula R. Kuser-Falcão, Michel E. B. Yamagishi, Edgard H. Santos, Fábio D. Vieira, José G. Jardine, and Goran Neshich. Sting_rdb: A relational database of structural parameters for protein analysis with support for data warehousing and data mining. *Genetics and Molecular Research*, 6(4):911–922, 2007.
5. Goran Neshich, Adauto L. Mancini, Michel E. B. Yamagishi, Paula R. Kuser, Renato Fileto, Ivan P. Pinto, Juliana F. Palandrani, João N. Krauchenco, Christian Baudet, Arnaldo J. Montagner, and Roberto H. Higa. Sting report: convenient web-based application for graphic and tabular presentations of protein sequence, structure and function descriptors from the sting database. *Nucleic Acids Research*, 33(Database issue):D269–D274, 2005.
6. Adauto L. Mancini, Roberto H. Higa, A. G. Oliveira, Paula R. Kuser, et al. Sting contacts: a web-based application for identification and analysis of amino acid contacts within protein structure and across protein interfaces. *Bioinformatics*, 20(13):2145–2147, 2004.
7. Fábio R. de Moraes, Izabella A. P. Neshich, Ivan Mazoni, Inácio H. Yano, José G. C. Pereira, José A. Salim, José G. Jardine, and Goran Neshich. Improving predictions of protein-protein interfaces by combining amino acid-specific classifiers based on structural and physicochemical descriptors with their weighted neighbor averages. *PLoS ONE*, 9(1):e87107, 2014.
8. Ivan Mazoni et al. Study of specific nanoenvironments containing α-helices in all-α and $(\alpha + \beta) + (\alpha/\beta)$ proteins. *PLoS ONE*, 13(5):e0197095, 2018.
9. Goran Neshich, Ivan Mazoni, S. Oliveira, Michel E. B. Yamagishi, Paula R. Kuser-Falcão, Luiz C. Borro, et al. The star sting server: a multiplatform

environment for protein structure analysis. *Genetics and Molecular Research*, 5:717–722, 2006. Accessed November 20, 2023; available at multiple URLs: http://sms.cbi.cnptia.embrapa.br/SMS/, http://trantor.bioc.columbia.edu/SMS/, http://www.es.embnet.org/SMS/, http://gibk26.bse.kyutech.ac.jp/SMS/, and http://www.ar.embnet.org/SMS/.

10. H. J. Lee, P. T. Lang, S. M. Fortune, C. M. Sassetti, and T. Alber. Cyclic AMP regulation of protein lysine acetylation in Mycobacterium tuberculosis. *Nature Structural & Molecular Biology*, 19(8):811–818, 2012.
11. D. J. Rigden, S. E. Phillips, P. A. Michels, and L. A. Fothergill-Gilmore. The structure of pyruvate kinase from Leishmania mexicana reveals details of the allosteric transition and unusual effector specificity. *Journal of Molecular Biology*, 291(3):615–635, 1999. Published correction appears in J Mol Biol 1999 Oct 29;293(3):745–749.
12. W. Huang, S. Lu, Z. Huang, et al. Allosite: a method for predicting allosteric sites. *Bioinformatics*, 29(18):2357–2359, 2013.
13. V Le Guilloux, P Schmidtke, and P Tuffery. Fpocket: an open source platform for ligand pocket detection. *BMC Bioinformatics*, 10:1–11, 2009.
14. Thomas A. Halgren. Identifying and characterizing binding sites and assessing druggability. *Journal of Chemical Information and Modeling*, 49(2):377–389, 2 2009.
15. Min Huang, Kun Song, Xinyi Liu, Shaoyong Lu, Qiancheng Shen, Renxiao Wang, Jingze Gao, Yuanyuan Hong, Qian Li, Duan Ni, Jianrong Xu, Guoqiang Chen, and Jian Zhang. AlloFinder: A strategy for allosteric modulator discovery and allosterome analyses. *Nucleic Acids Research*, 46(W1):W451–W458, 7 2018.
16. J. G. Greener and M. J. Sternberg. Allopred: prediction of allosteric pockets on proteins using normal mode perturbation analysis. *BMC Bioinformatics*, 16:335, 2015.
17. Alejandro Panjkovich and Xavier Daura. PARS: a web server for the prediction of Protein Allosteric and Regulatory Sites. *Bioinformatics*, 30(9):1314–1315, 5 2014.
18. C. R. Schwantes, R. T. McGibbon, and V. S. Pande. Perspective: Markov models for long-timescale biomolecular dynamics. *Journal of Chemical Physics*, 141(9), 9 2014.
19. H. Tian, X. Jiang, and P. Tao. Passer: Prediction of allosteric sites server. *Machine Learning: Science and Technology*, 2(3):035015, 2021. Epub 2021 May 13; PMID: 34396127; PMCID: PMC8360383.
20. Hao Tian, Sian Xiao, Xi Jiang, and Peng Tao. PASSer: fast and accurate prediction of protein allosteric sites. *Nucleic Acids Research*, 51(W1):W427–W431, 7 2023.
21. Moaaz Khokhar, Ozlem Keskin, and Attila Gursoy. DeepAllo: allosteric site prediction using protein language model (pLM) with multitask learning. *Bioinformatics*, 41(6), 6 2025.
22. Nadezhda T. Doncheva, Karsten Klein, Francisco S. Domingues, and Mario Albrecht. Analyzing and visualizing residue networks of protein structures. *Trends in Biochemical Sciences*, 36(4):179–182, 4 2011.
